# Exploration of Primary Care Clinician Attitudes and Cognitive Characteristics Associated With Prescribing Antibiotics for Asymptomatic Bacteriuria

**DOI:** 10.1001/jamanetworkopen.2022.14268

**Published:** 2022-05-27

**Authors:** Jonathan D. Baghdadi, Deborah Korenstein, Lisa Pineles, Laura D. Scherer, Alison D. Lydecker, Larry Magder, Deborah N. Stevens, Daniel J. Morgan

**Affiliations:** 1 Department of Epidemiology and Public Health, University of Maryland, Baltimore; 2 Veterans Affairs (VA) Maryland Healthcare System, Baltimore; 3 Division of General Internal Medicine, Memorial Sloan Kettering Cancer Center, New York, New York; 4 Adult and Child Consortium of Health Outcomes Research and Delivery Science, University of Colorado School of Medicine, Aurora; 5 Division of Cardiology, University of Colorado School of Medicine, Aurora; 6 Center of Innovation for Veteran-Centered and Value-Driven Care, VA Denver, Denver, Colorado

## Abstract

**Importance:**

Antibiotic treatment for asymptomatic bacteriuria is not recommended in guidelines but is a major driver of inappropriate antibiotic use.

**Objective:**

To evaluate whether clinician culture and personality traits are associated with a predisposition toward inappropriate prescribing.

**Design, Setting, and Participants:**

This survey study involved secondary analysis of a previously completed survey. A total of 723 primary care clinicians in active practice in Texas, the Mid-Atlantic, and the Pacific Northwest, including physicians and advanced practice clinicians, were surveyed from June 1, 2018, to November 26, 2019, regarding their approach to a hypothetical patient with asymptomatic bacteriuria. Clinician culture was represented by training background and region of practice. Attitudes and cognitive characteristics were represented using validated instruments to assess numeracy, risk-taking preferences, burnout, and tendency to maximize care. Data were analyzed from November 8, 2021, to March 29, 2022.

**Interventions:**

The survey described a male patient with asymptomatic bacteriuria and changes in urine character. Clinicians were asked to indicate whether they would prescribe antibiotics.

**Main Outcomes and Measures:**

The main outcome was self-reported willingness to prescribe antibiotics for asymptomatic bacteriuria. Willingness to prescribe antibiotics was hypothesized to be associated with clinician characteristics, background, and attitudes, including orientation on the Medical Maximizer-Minimizer Scale. Individuals with a stronger orientation toward medical maximizing prefer treatment even when the value of treatment is ambiguous.

**Results:**

Of the 723 enrolled clinicians, 551 (median age, 32 years [IQR, 29-44 years]; 292 [53%] female; 296 [54%] White) completed the survey (76% response rate), including 288 resident physicians, 202 attending physicians, and 61 advanced practice clinicians. A total of 303 respondents (55%) were from the Mid-Atlantic, 136 (25%) were from Texas, and 112 (20%) were from the Pacific Northwest. A total of 392 clinicians (71% of respondents) indicated that they would prescribe antibiotic treatment for asymptomatic bacteriuria in the absence of an indication. In multivariable analyses, clinicians with a background in family medicine (odds ratio [OR], 2.93; 95% CI, 1.53-5.62) or a high score on the Medical Maximizer-Minimizer Scale (indicating stronger medical maximizing orientation; OR, 2.06; 95% CI, 1.38-3.09) were more likely to prescribe antibiotic treatment for asymptomatic bacteriuria. Resident physicians (OR, 0.57; 95% CI, 0.38-0.85) and clinicians in the Pacific Northwest (OR, 0.49; 95% CI, 0.33-0.72) were less likely to prescribe antibiotics for asymptomatic bacteriuria.

**Conclusions and Relevance:**

The findings of this survey study suggest that most primary care clinicians prescribe inappropriate antibiotic treatment for asymptomatic bacteriuria in the absence of risk factors. This tendency is more pronounced among family medicine physicians and medical maximizers and is less common among resident physicians and clinicians in the US Pacific Northwest. Clinician characteristics should be considered when designing antibiotic stewardship interventions.

## Key Points

**Question:**

Are certain clinician attitudes and characteristics associated with unnecessary antibiotic prescribing for asymptomatic bacteriuria?

**Findings:**

In this survey study of 551 primary care clinicians, 392 (71%) reported that they would prescribe antibiotics for asymptomatic bacteriuria. Prescribing antibiotics for asymptomatic bacteriuria was more common among medical maximizers and family medicine physicians and was less common among resident physicians and clinicians in the US Pacific Northwest.

**Meaning:**

Clinician characteristics are associated with the decision to prescribe antibiotics and should be considered when designing antibiotic stewardship interventions.

## Teaser

This survey study assesses whether certain attitudes and characteristics of primary care clinicians are associated with a predisposition to prescribe antibiotics for asymptomatic bacteriuria.

## Introduction

In the absence of specific risk factors, antibiotic treatment for asymptomatic bacteriuria is not indicated.^1^ However, a positive culture is a strong stimulus to prescribe, and antibiotic treatment of asymptomatic bacteriuria remains common in apparent defiance of guidelines (including recommendations from the Infectious Diseases Society of America for the Choosing Wisely campaign).^2,3,4^ Across studies, approximately 45% of patients with asymptomatic bacteriuria receive inappropriate antibiotics.^5^ Inappropriate antibiotics in this context may cause harm through adverse drug events, *Clostridioides difficile* infection, or antibiotic resistance, but it will not provide benefit.^6,7,8,9^

Inappropriate antibiotic prescribing for asymptomatic bacteriuria is more common among patients with older age, female sex, history of dementia, acute altered mental status, abnormal urinalysis results, or peripheral leukoyctosis.^5,10^ In some cases, inappropriate prescribing may stem from diagnostic uncertainty, as when the patient cannot provide a clear symptom history or when serum studies demonstrate an unexplained peripheral leukocytosis. However, the association between inappropriate prescribing and abnormalities on urinalysis, including pyuria, hematuria, or the presence of nitrites, suggests that clinicians may treat inappropriately because they misinterpret the meaning of common diagnostic test results. Changes in urine character, except visible hematuria, are not symptoms consistent with urinary tract infection (UTI).^11,12^ Pyuria cannot differentiate between UTI and asymptomatic bacteriuria.^13,14^

Clinicians have cited multiple reasons for inappropriate prescribing, including a desire to meet their patient’s perceived expectations, a sense that evidence-based care does not always translate to the bedside, and a tension between individualized care and broader public health concerns.^15,16^ However, patients and clinicians may not always bring the same expectations to the health care interaction.^17,18^ Instead, through their interaction, the patient and clinician are likely taking cues from one another about whether antibiotics are expected.^19^ The interplay between patient and clinician expectations is one example of how clinician characteristics can influence medical decision-making.^20^

Identifying clinician traits associated with inappropriate prescribing could inform targeted antibiotic stewardship interventions to preemptively address antibiotic overuse. This study sought to explore whether clinician attitudes and cognitive characteristics inform the decision to prescribe inappropriate antibiotics for asymptomatic bacteriuria. Specifically, we hypothesized that numeracy may influence antibiotic prescribing by distorting clinician understanding of probability of illness.^21,22^ Burnout may limit clinicians’ willingness to engage their patients in a difficult conversation about resistance.^23^ Tolerance of risk and uncertainty may influence a clinician’s likelihood of practicing defensive prescribing.^24,25^ Tendency to maximize medical care can predispose clinicians toward unnecessary prescribing.^26^

## Methods

We conducted a survey between June 1, 2018, and November 26, 2019, of 723 primary care clinicians in Delaware, Maryland, Oregon, Pennsylvania, Texas, Virginia, Washington, and the District of Columbia to explore their understanding of and reactions to common diagnostic tests. Development of the survey and recruitment of the sample are described in detail elsewhere.^21^ Clinicians were identified and enrolled during site visits to approximately 30 eligible clinics. Completion of the survey was incentivized with a US $50 gift card. Institutional review board approval was obtained at each of 3 coordinating sites (Baltimore, Maryland; San Antonio, Texas; and Portland, Oregon). Verbal informed consent with a waiver of documentation was approved at all sites to protect confidentiality. The study followed the American Association for Public Opinion Research (AAPOR) reporting guideline.

The survey presented 4 clinical scenarios, including 1 case of asymptomatic bacteriuria:

“Mr. Williams, a 65-year-old man, comes to the office for follow-up of his osteoarthritis. He has noted foul-smelling urine and no pain or difficulty with urination. A urine dipstick shows trace blood. He has no particular preference for testing and wants your advice.”

Respondents were prompted to indicate whether they would prescribe antibiotics. They were also asked to estimate the probability that the patient described in the clinical scenario had a UTI: “How likely is Mr. Williams to have a urinary tract infection (UTI)?”

Survey items included validated instruments to assess numeracy, burnout, comfort with uncertainty, tendency toward risk-taking, fear of malpractice, and tendency toward medical maximalism as well as demographic information and training background.^22,23,24,27,28^ Race and ethnicity were selected by participants from a list of categories. Medical maximalism represents clinician and patient preferences regarding active and passive approaches to health care use. Orientation toward medical maximizing was assessed using a modified version of the Medical Maximizer-Minimizer Scale (eAppendix in the Supplement), which includes prompts that query whether “it is important to treat a disease even when it does not make a difference in quality of life.” Individuals with a stronger orientation toward medical maximizing prefer treatment even when the value of treatment is ambiguous.

### Statistical Analysis

Survey data were entered into REDCap (Research Electronic Data Capture; Vanderbilt University Medical Center).^22,24,27,28,29^ Associations between clinician characteristics and treatment of asymptomatic bacteriuria were measured using the χ^2^ test or Fisher exact test, as appropriate, for categorical variables and the Wilcoxon rank sum tests for continuous variables. Multivariable models used logistic regression to evaluate the association between clinician character traits and likelihood of prescribing after controlling for clinician background. All statistical tests were 2-tailed with α = .05. Statistical analyses were performed from November 8, 2021, to March 29, 2022, with Stata software, version 14.2/IC (StataCorp LLC).

## Results

### Participant Demographic Characteristics

A total of 723 primary care physicians, nurse practitioners, and physician assistants were offered the survey, 585 individuals responded, and 551 answered all questions (76% complete response rate), including 288 resident physicians, 202 attending physicians, and 61 advanced practice clinicians. The median age of respondents was 32 years (IQR, 29-44 years). A total of 291 respondents (53%) were female and 258 (47%) were male (*P* = .08). A total of 142 respondents (26%) were Asian/Pacific Islander, 37 (7%) were Black, 45 (8%) were Latinx, 296 (54%) were White, and 19 (3%) were more than 1 race; race was not specified for 12 (2%) (*P* = 0.34). Respondents had varying levels of experience, including 238 clinicians (43% of the sample) who were in practice for less than 3 years, 160 (29%) who were in practice for 3 to 9 years, and 145 (26%) who were in practice for 10 or more years. Respondents predominantly practiced in Baltimore and the Mid-Atlantic region (303 of 551 [55%]) followed by San Antonio and Texas (136 of 551 [25%]) and Portland and the Pacific Northwest (112 of 551 [20%]). Only 61 respondents (11%) were advanced practice clinicians (nurse practitioners or physician assistants).

### Training and Professional Background of Clinicians Who Would Treat Asymptomatic Bacteriuria With Antibiotics

Overall, 392 of the 551 respondents (71%) who completed the survey indicated that they would prescribe antibiotic treatment for the patient with asymptomatic bacteriuria described in the scenario (Table 1). The subgroups most likely to prescribe antibiotics for asymptomatic bacteriuria were clinicians in practice for at least 10 years (119 [82%] would prescribe vs 116 clinicians [73%] with 3-9 years in practice vs 153 [64%] of clinicians with <3 years in practice; *P* = .001), clinicians with a background in family medicine (120 [85%] vs 207 [62%] in internal medicine; *P* < .001), advanced practice clinicians (55 [90%] vs 157 [78%] of attending physicians vs 180 [63%] resident physicians; *P* < .001), and clinicians who had previously been sued for malpractice (28 [90%] vs 363 [70%] who had not; *P* = .02). However, regardless of years in practice, training background, or professional degree, most clinicians indicated that they would prescribe antibiotics for asymptomatic bacteriuria.

**Table 1.** Associations of Treatment of Asymptomatic Bacteriuria With Antibiotic With Clinician Characteristics on Bivariable Analysis

Characteristic	Treat asymptomatic bacteriuria with antibiotics, No. (%) (N = 551)		*P* value^a^
	Yes (n = 392)	No (n = 159)	
Age, y			
<30 (n = 171)	108 (63)	63 (37)	<.001
30-39 (n = 207)	142 (69)	65 (31)	
≥40 (n = 167)	136 (81)	31 (19)	
Study site			
Portland and Pacific Northwest (n = 112)	73 (65)	39 (35)	.15
Baltimore and Mid-Atlantic (n = 303)	215 (71)	88 (29)	
San Antonio and Texas (n = 136)	104 (76)	32 (24)	
Degree and training			
MD or DO resident (n = 288)	180 (63)	108 (38)	<.001
MD or DO attending (n = 202)	157 (78)	45 (22)	
NP or PA (n = 61)	55 (90)	6 (10)	
Specialty (MDs and DOs)			
Internal medicine (n = 335)	207 (62)	128 (38)	<.001
Family medicine (n = 142)	120 (85)	22 (15)	
Other (n = 75)	65 (17)	9 (6)	
Time in practice since graduation, median (IQR), y			
<3 (n = 238)	153 (64)	85 (36)	.001
3-9 (n = 160)	116 (73)	44 (28)	
≥10 (n = 145)	119 (82)	26 (18)	
Ever sued for malpractice			
Yes (n = 31)	28 (90)	3 (10)	.02
No (n = 519)	363 (70)	156 (30)	
Numeracy score (range, 0-3)			
Median (IQR)	3 (2-3)	3 (2-3)	.008
Low (score of 0-1 of 3) (n = 64)	50 (78)	14 (22)	.03
Medium (score of 2 of 3) (n = 172)	131 (76)	41 (24)	
High (score of 3 of 3) (n = 305)	202 (66)	103 (34)	
Medical Maximizer-Minimizer Scale score (range, 1-7)			
Median (IQR)	3.00 (2.29-3.57)	2.57 (2.00-3.29)	.003
Low (score <2.4) (n = 169)	108 (64)	60 (36)	.04
Medium (score of 2.3-3.39) (n = 212)	153 (72)	59 (28)	
High (score ≥3.4) (n = 164)	126 (77)	38 (23)	
Risk-taking score (range, 6-30, with higher scores indicating risk seeking), median (IQR)	17 (14-21)	17 (14-21)	.84
Fear of malpractice (range, 6-30), median (IQR)^b^	17 (13-20)	16 (13-20)	.58
Burnout score (range, 1-5), median (IQR)^c^	2 (2-3)	2 (2-3)	.34
The Revised Physicians’ Reactions to Uncertainty subscale scores, median (IQR)			
Often uncertain in medical practice (range, 1-6)^d^	5 (4-6)	5 (5-6)	.04
Stress from uncertainty (range, 3-18)^e^	11 (9-12)	10 (8-12)	.30
Concern about bad outcomes (range, 3-18)^f^	10 (8-13)	10 (8-12)	.69
Perceived likelihood of patient having a UTI, median (IQR), % probability	90 (80-100)	15 (5-30)	<.001

Abbreviations: DO, doctor of osteopathy; MD, doctor of medicine; NP, nurse practitioner; PA, physician assistant; UTI, urinary tract infection.

^a^ Test statistic from χ^2^ or Fisher exact test, as appropriate, for categorical variables and from Wilcoxon rank sum tests for continuous variables.

^b^ Higher scores indicate greater fear of malpractice.

^c^ Higher scores indicate a greater degree of burnout.

^d^ In response to the statement “There is often uncertainty in medical practice,” a score of 6 indicates strong agreement, and 1 indicates strong disagreement.

^e^ Higher scores indicate greater stress from uncertainty.

^f^ Higher scores indicate greater concern about bad outcomes.

### Cognitive Characteristics of Clinicians Who Would Treat Asymptomatic Bacteriuria With Antibiotics

In unadjusted analyses, having a low numeracy score (50 [78%] vs 14 [22%]; *P* = .03) and a stronger medical maximizing orientation (score of 3.00 [IQR, 2.29-3.57] vs 2.57 [IQR, 2.00-3.29]; *P* < .003) were associated with increased reported willingness to prescribe vs not prescribe antibiotics for asymptomatic bacteriuria. Conversely, clinicians who indicated recognition of uncertainty in the practice of medicine on the Revised Physicians’ Reactions to Uncertainty subscale were less likely to prescribe antibiotics for asymptomatic bacteriuria (median subscale score, 5 [IQR, 5-6] vs 5 [IQR, 4-6]; *P* = .04). The decision to prescribe antibiotics was not associated with fear of malpractice, level of burnout, or stress from uncertainty.

### Multivariable Analyses

Clinician characteristics were evaluated using multivariable analysis to identify independent factors associated with antibiotic prescribing for asymptomatic bacteriuria (Table 2). In adjusted analyses, clinicians in Portland and the Pacific Northwest (odds ratio [OR], 0.49; 95% CI, 0.33-0.72) were less likely than clinicians in other regions to prescribe antibiotics for asymptomatic bacteriuria. Resident physicians (OR, 0.57; 95% CI, 0.38-0.85) were less likely to prescribe than attending physicians, and clinicians with a training background in family medicine (OR, 2.93; 95% CI, 1.53-5.62) were more likely than internists to prescribe. A high score on the Medical Maximizer-Minimizer Scale (indicating stronger medical maximizing orientation) was associated with increased likelihood of prescribing antibiotics for asymptomatic bacteriuria (OR, 2.06; 95% CI, 1.38-3.09) compared with a low score. Numeracy scores were not significantly associated with prescribing.

**Table 2.** Results From Multilevel Models of Associations Between Clinician Characteristics and Reported Treatment of Asymptomatic Bacteriuria With Antibiotics^a^

Variable	Parsimonious model including numeracy score and Medical Maximizer-Minimizer Scale		Expanded model including all scales	
	OR (95% CI)	*P* value	OR (95% CI)	*P* value
Years in practice	1.01 (0.99-1.03)	.41	1.01 (0.99-1.03)	.31
Resident physician	0.57 (0.38-0.85)	.006	0.58 (0.43-0.79)	<.001
NP or PA	2.30 (0.85-6.24)	.10	2.49 (0.80-7.74)	.12
Specialty				
Internal medicine	1 [Reference]	NA	1 [Reference]	NA
Family medicine	2.93 (1.53-5.62)	.001	3.09 (1.68-5.68)	<.001
Other specialty	1.73 (0.83-3.60)	.14	1.61 (0.70-3.73)	.26
Study site				
Baltimore and Mid-Atlantic	1 [Reference]	NA	1 [Reference]	NA
Portland and Pacific Northwest	0.49 (0.33-0.72)	<.001	0.52 (0.34-0.81)	.003
San Antonio and Texas	0.97 (0.97-0.98)	<.001	1.06 (1.03-1.08)	<.001
Previously sued for malpractice	2.09 (0.65-6.70)	.22	2.27 (0.71-7.23)	.17
Numeracy score				
Low	1 [Reference]	NA	1 [Reference]	NA
Medium	1.14 (0.66-1.96)	.64	1.09 (0.64-1.85)	.76
High	0.79 (0.50-1.24)	.31	0.78 (0.51-1.19)	.24
Medical Maximizer-Minimizer Scale score				
Low	1 [Reference]	NA	1 [Reference]	NA
Medium	1.63 (1.30-2.05)	<.001	1.72 (1.32-2.25)	<.001
High	2.06 (1.38-3.09)	<.001	2.13 (1.39-3.24)	<.001
Risk-taking score	NA	NA	1.01 (0.97-1.06)	.53
Fear of malpractice score	NA	NA	1.01 (0.98-1.04)	.72
Burnout score	NA	NA	1.11 (0.99-1.24)	.07
Stress from uncertainty subscale	NA	NA	1.04 (1.00-1.07)	.06

Abbreviations: NA, not applicable; NP, nurse practitioner; OR, odds ratio; PA, physician assistant.

^a^ Models were specified using multilevel mixed-effects logistic regression, including random intercepts for study site.

### Estimates of Probability of UTI

On average, respondents who would prescribe antibiotics for the case patient estimated a 90% probability of UTI (95% CI, 80%-100%). Respondents who would not prescribe antibiotics estimated that the patient had a 15% probability of UTI (95% CI, 5%-30%).

## Discussion

In this study of more than 500 primary care clinicians across 7 states and the District of Columbia, 7 of 10 clinicians reported that they would prescribe inappropriate antibiotics for asymptomatic bacteriuria. This tendency was most pronounced among attending physicians, family medicine physicians, and clinicians outside the Pacific Northwest. However, most clinicians, regardless of degree type, years in practice, or geographic region, reported being willing to prescribe inappropriate antibiotics. These findings suggest that the Choosing Wisely campaign recommending against antibiotic treatment for asymptomatic bacteriuria^3^ has failed to make an impact in the US and that certain types of clinicians are more likely than others to ignore the guidelines and prescribe antibiotics.

In some instances, willingness to prescribe inappropriate antibiotics among our study sample likely reflects a knowledge gap.^4^ Overwhelmingly, clinicians who indicated they would prescribe antibiotics estimated that the patient had a high probability of having a UTI, although the case details did not support this diagnosis. We suspect that many clinicians in our sample were not aware of what constitutes UTI symptoms or were not aware that symptoms are required to substantiate a UTI diagnosis. For attending physicians, these knowledge gaps may have been perpetuated by ambiguity in the peer-reviewed literature encountered during their training. Although guidelines state that changes in urine character are nonspecific,^1^ older studies sometimes classify changes in urine character as a symptom.^30^ Given that current residents were less likely than attending physicians to prescribe antibiotics, greater clarity in the recent literature on what constitutes a symptom and evolving graduate medical education on appropriate management of asymptomatic bacteriuria may mean that knowledge gaps will be less of an issue moving forward.

However, knowledge gaps alone likely do not explain the observed tendency toward inappropriate antibiotic prescribing. Differences in health care culture drive variation in patterns of use between geographic regions.^31^ We observed regional variation that suggests cultural factors also influence use of inappropriate antibiotics. In our sample, clinicians in Portland and the Pacific Northwest were less likely to prescribe antibiotics for asymptomatic bacteriuria than clinicians in other regions. We suspect this finding illustrates a culture of high-value care in this part of the country, as was previously demonstrated by the *Dartmouth Atlas of Health Care*.^32^

Finally, in addition to knowledge and culture, clinician attitudes and cognitive characteristics were associated with the decision to prescribe inappropriate antibiotics for asymptomatic bacteriuria. We found that clinicians who had a stronger medical maximizing (vs minimizing) orientation were more likely to prescribe inappropriate antibiotics. Medical maximizers favor errors of commission over errors of omission, preferring to treat even when treatment has uncertain value and may introduce a chance of harm. Our finding of an association between medical maximizing and inappropriate antibiotic prescribing is important because it suggests that certain tendencies among clinicians may pose a barrier to initiatives, such as Choosing Wisely, that are intended to combat the emergence of antimicrobial resistance.

Further study is needed to determine whether an understanding of the medical maximizer-minimizer orientation of target clinicians can inform more effective interventions to reduce inappropriate antibiotic prescribing for asymptomatic bacteriuria. We suspect that medical maximizers may be more resistant than their peers to interventions designed to reduce low-value care if low value is defined by low likelihood of providing benefit.^33^ Instead, interventions that target medical maximizers may be more effective if they emphasize avoiding harm.^34^ For example, an intervention that ranks clinicians on frequency of inappropriate antibiotic prescriptions could be framed as a list of clinicians most frequently prescribing potentially harmful treatment rather than clinicians most frequently prescribing unnecessary treatment.

### Limitations

Our study is subject to the limitations of a survey conducted to assess responses to a hypothetical patient. Most important, the survey included a single clinical scenario related to asymptomatic bacteriuria and, as such, cannot reflect the myriad ways that asymptomatic bacteriuria may present among real patients. Respondents’ reported tendencies toward antibiotic prescribing may not match their actual practice when faced with a living patient. In addition, although the response rate to the survey was high, the sample may not be sufficiently large to be adequately powered for multiple subgroup analyses.

## Conclusions

The results of this survey study suggest that most primary care clinicians would contradict widely accepted guidelines by prescribing antibiotics for asymptomatic bacteriuria in the absence of risk factors. This tendency was strongest among family medicine clinicians and medical maximizers and was less common among resident physicians and clinicians in the Pacific Northwest. Interventions designed in consideration of clinician culture, attitudes, and cognitive characteristics may more effectively reduce unnecessary treatment of asymptomatic bacteriuria.

## Notes

Supplement. **eAppendix.** Scales Included in Survey
