# Supplementary material for: Exploration of Primary Care Clinician Attitudes and Cognitive Characteristics Associated With Prescribing Antibiotics for Asymptomatic Bacteriuria
Source: JAMA Netw Open. 2022 May 27;5(5):e2214268. doi: 10.1001/jamanetworkopen.2022.14268 (PMC9142875; doi:10.1001/jamanetworkopen.2022.14268)
Supplement: Supplement. — eAppendix. Scales Included in Survey [file jamanetwopen-e2214268-s001.pdf]

Supplementary Online Content

Baghdadi JD, Korenstein D, Pineles L, et al. Exploration of primary care clinician attitudes and cognitive characteristics associated with prescribing antibiotics for asymptomatic bacteriuria. *JAMA Netw Open*. 2022;5(5):e2214268. doi:10.1001/jamanetworkopen.2022.14268

eAppendix. Scales Included in Survey

This supplementary material has been provided by the authors to give readers additional information about their work.

© 2022 Baghdadi JD et al. *JAMA Network Open.*

eAppendix. Scales Included in Survey

THE REVISED PHYSICIANS' REACTIONS TO UNCERTAINTY SCALES

Gerrity MS, White KP, DeVellis RF, Dittus. Physicians' reactions to uncertainty: refining the constructs and scales. Motivation and Emotion 1995;19:175-191.

Stress from Uncertainty Subscale

Final score is sum of scores for participants who answered all 3 questions (possible range 3-18). Higher score indicates higher stress from uncertainty.

Items are rated on a 6-point Likert scale: 1 = strongly disagree, 2 = moderately disagree, 3= slightly disagree, 4 = slightly agree, 5 = moderately agree, 6 = strongly agree.

**4 items (adapted version)**
There is often uncertainty in the practice of medicine
I find the uncertainty involved in patient care disconcerting.
Uncertainty in patient care makes me uneasy.
The uncertainty of patient care often troubles me.

Concern About Bad Outcomes Subscale

Final score is sum of scores for participants who answered all 3 questions (possible range 3-18). Higher score indicates higher concern about bad outcomes.

Items are rated on a 6-point Likert scale: 1 = strongly disagree, 2 = moderately disagree, 3 = slightly disagree, 4 = slightly agree, 5 = moderately agree, 6 = strongly agree.

**3 items**
When I am uncertain of a diagnosis, I imagine all sorts of bad scenarios - patient dies, patient sues, etc...
I fear being held accountable for the limits of my knowledge.
I worry about malpractice when I do not know a patient's diagnosis.

FEAR OF MALPRACTICE

Katz D, Williams G, Brown R, et al. Emergency Physicians' Fear of Malpractice in Evaluating Patients With Possible Acute Cardiac Ischemia

Final score is sum of responses for participants who answered all 6 questions (possible range 6-30). Higher scores indicate higher fear of malpractice.

Items are rated on a 5-point Likert scale: 1 = disagree strongly, 2 = disagree, 3= neutral, 4 = agree, 5 = agree strongly.

© 2022 Baghdadi JD et al. *JAMA Network Open.*

6 Items
1. I have had to make significant changes in my practice pattern because of recent legal developments concerning medical delivery.
2. I am concerned that I will be involved in a malpractice case sometime in the next 10 years.
3. I feel pressured in my day-to-day practice by the threat of malpractice litigation.
4. I order some tests or consultations simply to avoid the appearance of malpractice.
5. Sometimes I ask for consultant opinions primarily to reduce my risk of being sued.
6. Relying on clinical judgment rather than on technology to make a diagnosis is becoming riskier from a medicolegal perspective.

Medical Maximizer-Minimizer Scale (Modified Version)

Scherer LD, Caverly TJ, Burke J, Zikmund-Fisher BJ, Kullgren JT, Steinley D, McCarthy DM, Roney M, Fagerlin A. Development of the Medical Maximizer-Minimizer Scale. Health Psychol. 2016 Nov;35(11):1276-1287.

Final score is the mean of all items for participants who answered all 7 questions.

Higher scores indicate more maximizing preferences.

Items are rated on a 7-point Likert scale: 1 = disagree strongly, 7=agree strongly

8 items (adapted version)
1. It is important to treat disease even when it does not make a difference in survival
2. It is important to treat a disease even when it does not make a difference in quality of life
3. Doing everything to fight illness is always the right choice
4. When it comes to health care, the only responsible thing to do is to actively seek medical care
5. If I have a health issue, my preference is to wait and see if the problem gets better on its own before *doing anything about it**
6. When it comes to health care, watching and waiting is never an acceptable option
7. When it comes to medical treatment, more is usually better
8. Diagnostic tests always provide helpful information even if their results do not directly impact care

* Item is reverse scored

Risk Taking Scale

Pearson SD, Goldman L, Orav EJ, et al. Triage decisions for emergency department patients with chest pain: do physicians' risk attitudes make the difference? J Gen Intern Med. 1995;10(10):557–564.

Final risk taking score = sum of responses x (6-[6 - number of missing responses]).

© 2022 Baghdadi JD et al. *JAMA Network Open.*

Higher score indicates “risk seeking” and lower score indicates “risk aversion”.

Items are rated on a 6-point Likert scale: 1 = strongly disagree, 2 = moderately disagree, 3= slightly disagree, 4 = slightly agree, 5 = moderately agree, 6 = strongly agree

**6 items**
1. I enjoy taking risks.
2. I try to avoid situations that have uncertain outcomes.*
3. Taking risks does not bother me if the gains involved are high.
4. I consider security an important element in every aspect of my life.*
5. People have told me that I seem to enjoy taking chances.
6. I rarely, if ever, take risks when there is another alternative.*

* Item is reverse scored

NUMERACY

Schwartz LM, Woloshin S, Black WC, Welch HG. The role of numeracy in understanding the benefit of screening mammography. Ann Intern Med. 1997;127 (11):966-972.

A reliable, validated instrument frequently used in numeracy studies. Scored as the total number of correct responses (range 0-3).

**3 items**
1. Imagine that we flip a fair coin 1,000 times. What is your best guess as to how many times the coin would come up heads in 1,000 flips?
2. In the BIGS BUCKS LOTTERY, the chance of winning a $10 is 1%. What is your best guess as to how many people would win a $10 prize if 1000 people each buy a single ticket to BIG BUCKS LOTTERY?
3. In ACME PUBLISHING SWEEPSTAKES, the chance of winning a car is 1 in 1,000. What percent of tickets to ACME PUBLISHING SWEEPSTAKES win a car?

© 2022 Baghdadi JD et al. *JAMA Network Open.*
